# Supplementary material for: Field-induced ultrafast modulation of Rashba coupling at room temperature in ferroelectric α-GeTe(111)
Source: Nat Commun. 2022 Oct 27;13:6396. doi: 10.1038/s41467-022-33978-3 (PMC9613697; doi:10.1038/s41467-022-33978-3)
Supplement: Supplementary file 1 — Supplementary Information [file 41467_2022_33978_MOESM1_ESM.pdf]

Supplementary Information: Field-induced ultra fastmodulation of Rashba coupling at room temperature in ferroelectric α-GeTe(111)

G. Kremer,[*,1,2] J. Maklar,[3] L. Nicolaï,[4] C. W. Nicholson,[1,3] C. Yue,[1] C. Silva,[3] P.Werner,[1] J. H. Dil,[5,6] J. Krempaský,[5] G. Springholz,[7] R. Ernstorfer,[3,8] J. Minár,[4*] L. Rettig,[3] and C. Monney[1]

1 Département de Physique and Fribourg Center for Nanomaterials, Université de Fribourg, CH-1700 Fribourg, Switzerland

2 Université Paris-Saclay, CNRS, Centre de Nanosciences et de Nanotechnologies, 91120, Palaiseau, France

3 Fritz Haber Institute of the Max Planck Society, Faradayweg 4-6, 14195 Berlin, Germany

4 New Technologies-Research Center University of West Bohemia, Plzen, Czech Republic

5 Photon Science Division, Paul Scherrer Institut, CH-5232 Villigen, Switzerland

6 Institute of physics, Ecole Polytechnique Fédérale de Lausanne, CH-1015 Lausanne, Switzerland

7 Institut für Halbleiter-und Festkörperphysik, Johannes Kepler Universität, A-4040 Linz, Austria

8 Institut für Optik und Atomare Physik, Technische Universität Berlin, Straße des 17, Juni 135, 10632 Berlin, Germany

E-mail: geoffroy.kremer@universite-paris-saclay.fr; jminar@ntc.zcu.cz

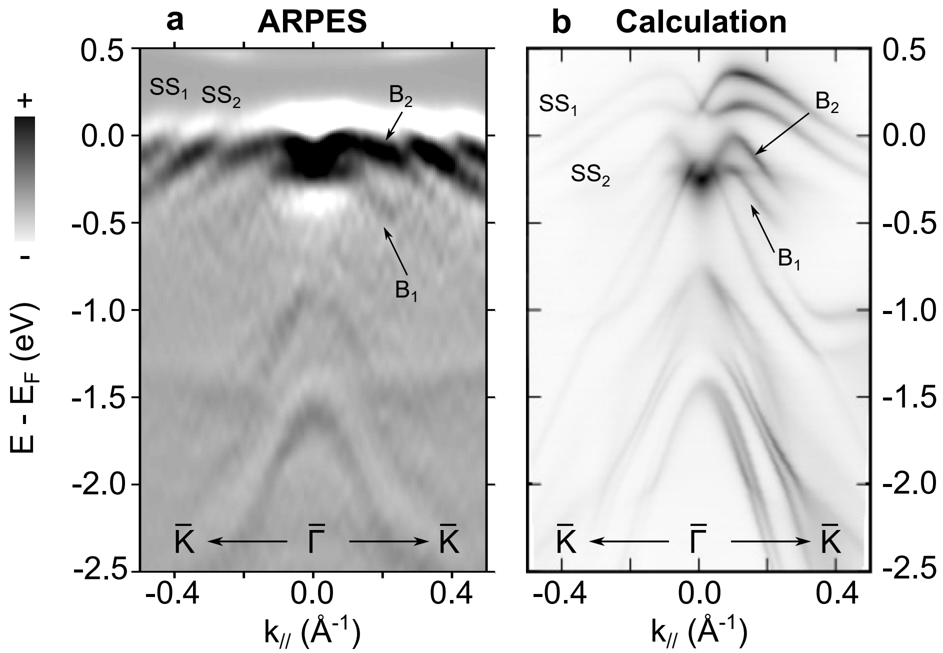

Supplementary Fig. 1: (a) Second derivative of static ARPES spectrum along the $\bar{K} - \bar{\Gamma} - \bar{K}$ high-symmetry direction and (b) corresponding one-step model of photoemission calculations for a Te-terminated surface of α-GeTe(111) with short surface bonds.

Second derivative of static α-GeTe(111) ARPES spectrum

Supplementary Fig. 1a shows the second derivative of ARPES spectrum in Fig. 1b in the main text. It better highlights the dispersion of Rashba-split bulk branches B_1 and B_2.

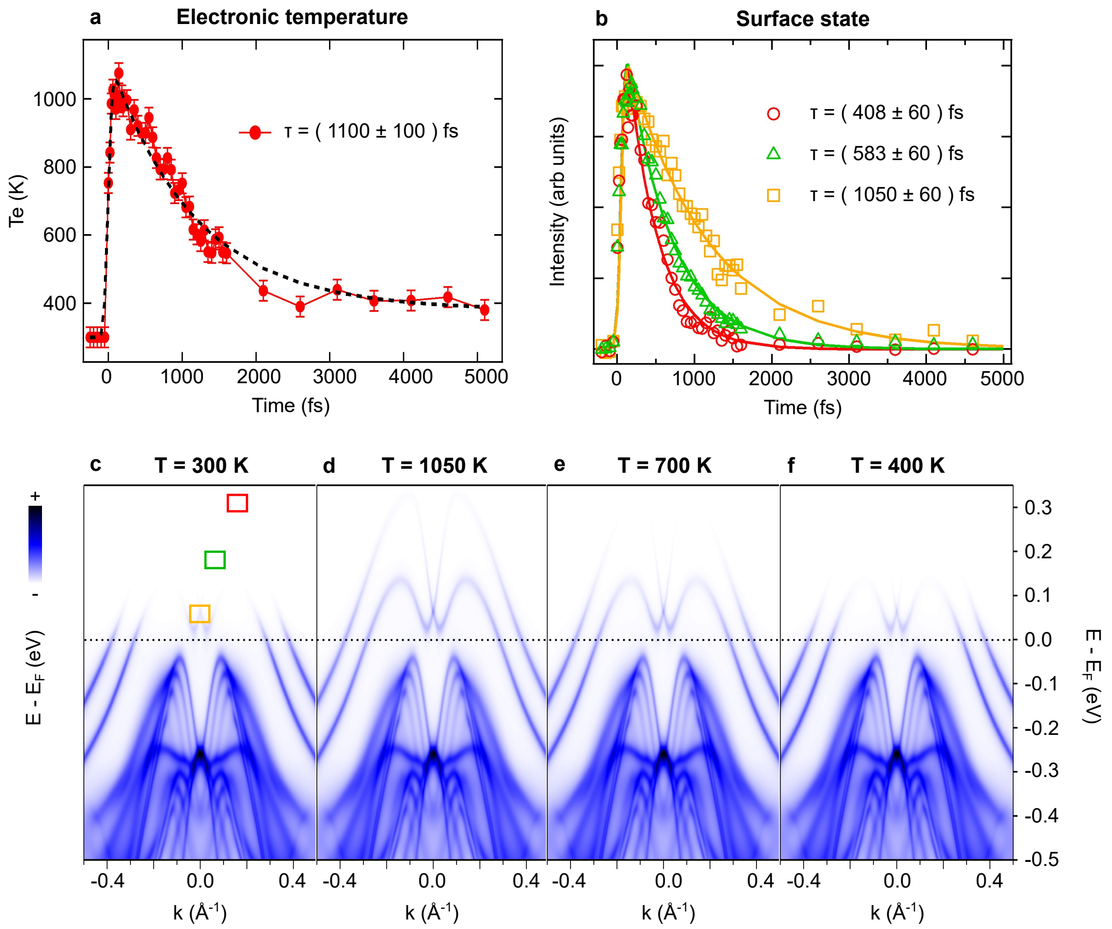

Supplementary Fig. 2: (a) Transient electronic temperature extracted by fitting the Fermi-Dirac distribution in the experimental data at different time delays for an absorbed fluence of 0.5 mJ/cm^2. (b) Transient photoemission intensity from experimental data integrated within the colored integration squares indicated in panel (c).(c,d,e,f) Bloch Spectral Function (BSF) band structure calculations of α-GeTe(111) multiplied by Fermi-Dirac distribution at different temperatures obtained from panel (a).

Thermal surface state population

Supplementary Fig. 2a shows the temporal evolution of the transient electronic temperature T_e as extracted by fitting the Fermi-Dirac distribution in the experimental data. It shows a maximum at 180 fs corresponding to $T_e = 1050$ K before slowly recovering with a time constant of 1.1 ps, as fitted with an exponential decay. This is a standard behaviour for a semiconducting material.[1] In Supplementary Fig. 2b we plot the transient evolution of the photoemission intensity at different positions along the surface state dispersion. The higher we are above E_F, the faster is the recovery time constant extracted from an exponential decay.

Nevertheless, the rise time is the same for each curve and their maximum correspond to 180 fs. This can be understood by plotting the calculated surface state dispersion convoluated by the Fermi-Dirac distribution at different temperatures, as shown in Supplementary Fig. 2c. The higher the temperature is, the more the surface state is populated above E_F. So we can conclude that the surface state is populated above E_F due to the transient increase of the electronic temperature. This is confirmed by comparing the orange curve in panel (b) and the electronic temperature from panel (a). The two curves perfectly fit.

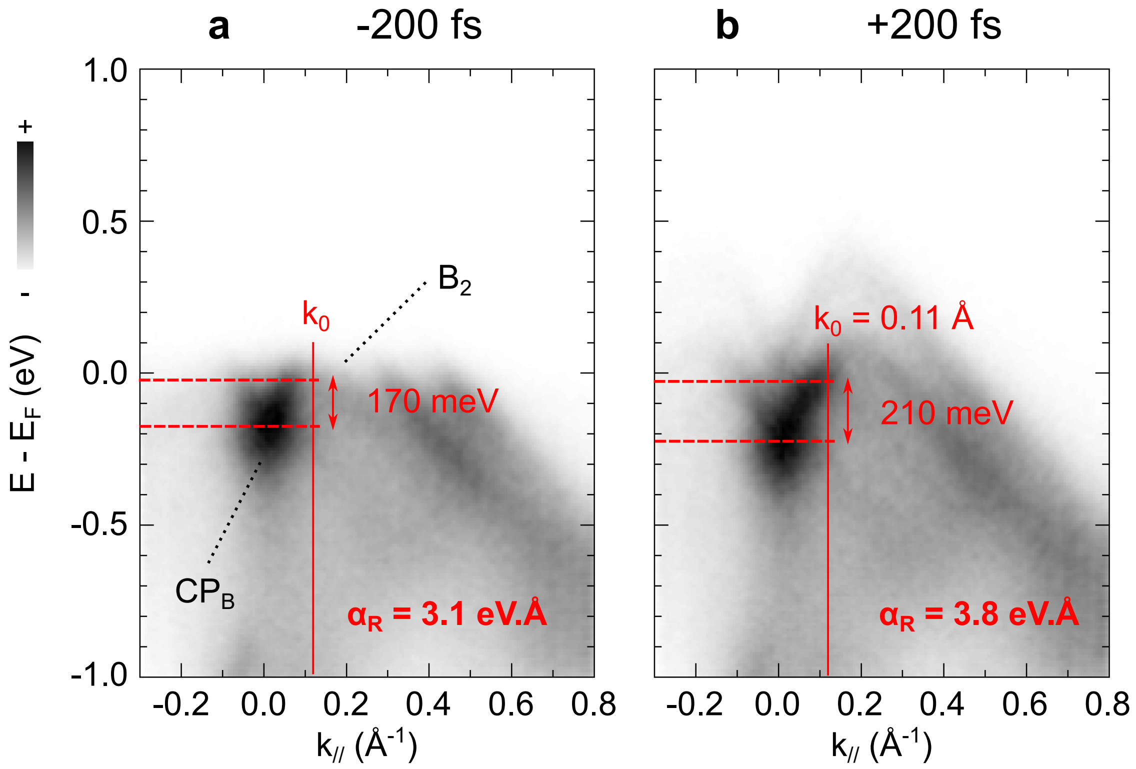

Supplementary Fig. 3: (a) tr-ARPES spectra of α-GeTe(111) recorded at pump-probe delays of (a) -200 fs and (b) $+200$ fs. The data have been acquired along the $\bar{K} - \bar{\Gamma} - \bar{K}$ direction with an absorbed pump fluence of 1 mJ/cm^2.

Evaluation of the Rashba parameter

Supplementary Fig. 3 shows the transient evolution of the Rashba parameter of the bulk bands at time delays of -200 fs and 200 fs as extracted using the standard relation $\alpha_R = 2E_R/k_0$.

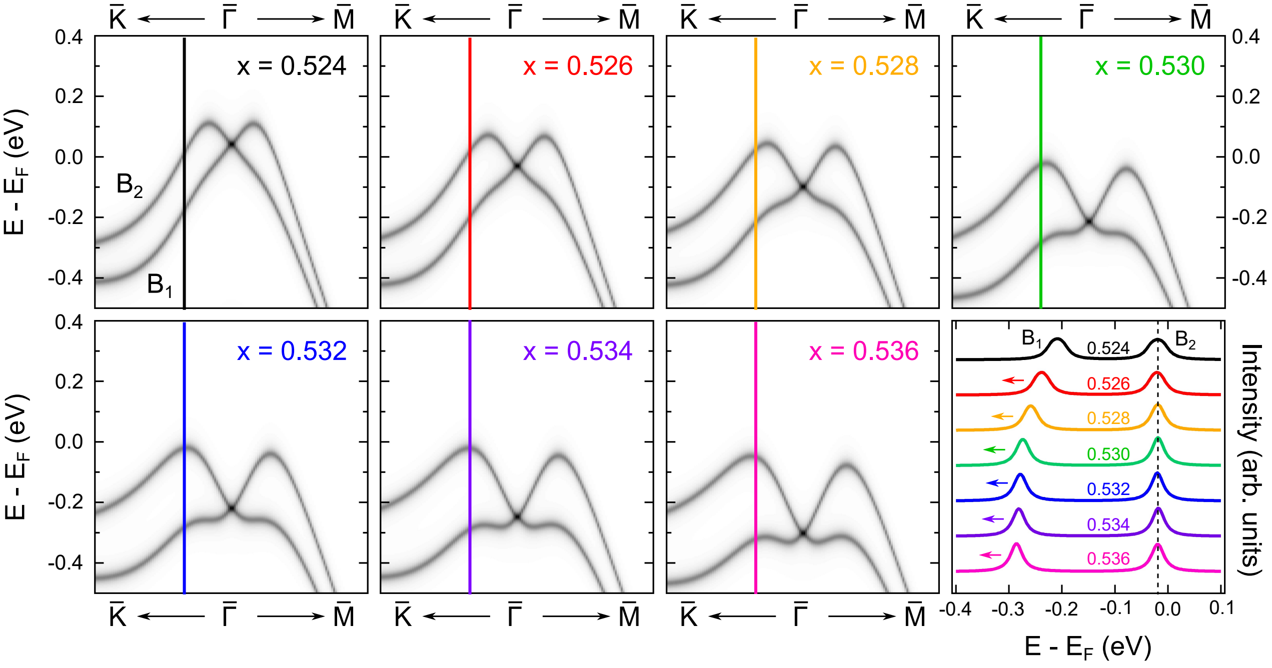

Supplementary Fig. 4: Band structure calculation of α-GeTe(111) for a series of x positions of the Te atom in the primitive unit cell, as defined in the inset of Fig. 3d. The ground state corresponds to x = 0.530. Bottom right panel shows energy distribution curves (EDCs) aligned relatively to the B_2 contribution away from the $\bar{\Gamma}$ point (see vertical colored lines in other panels).

Band structure and ARPES calculations as a function of the ferroelectric distorsion

Supplementary Fig. 4 shows band structure calculations of α-GeTe(111) for a series of x positions of the Te atom in the primitive unit cell. The more the value of x deviates from 0.5, corresponding to the centrosymmetric paraelectric phase, the more the Rashba splitting is important. This is illustrated in the bottom right panel. The B_2 contribution has been fixed to the position for x = 0.524 and the B_1 contribution shifts to higher binding energy (BE) when x is increasing.

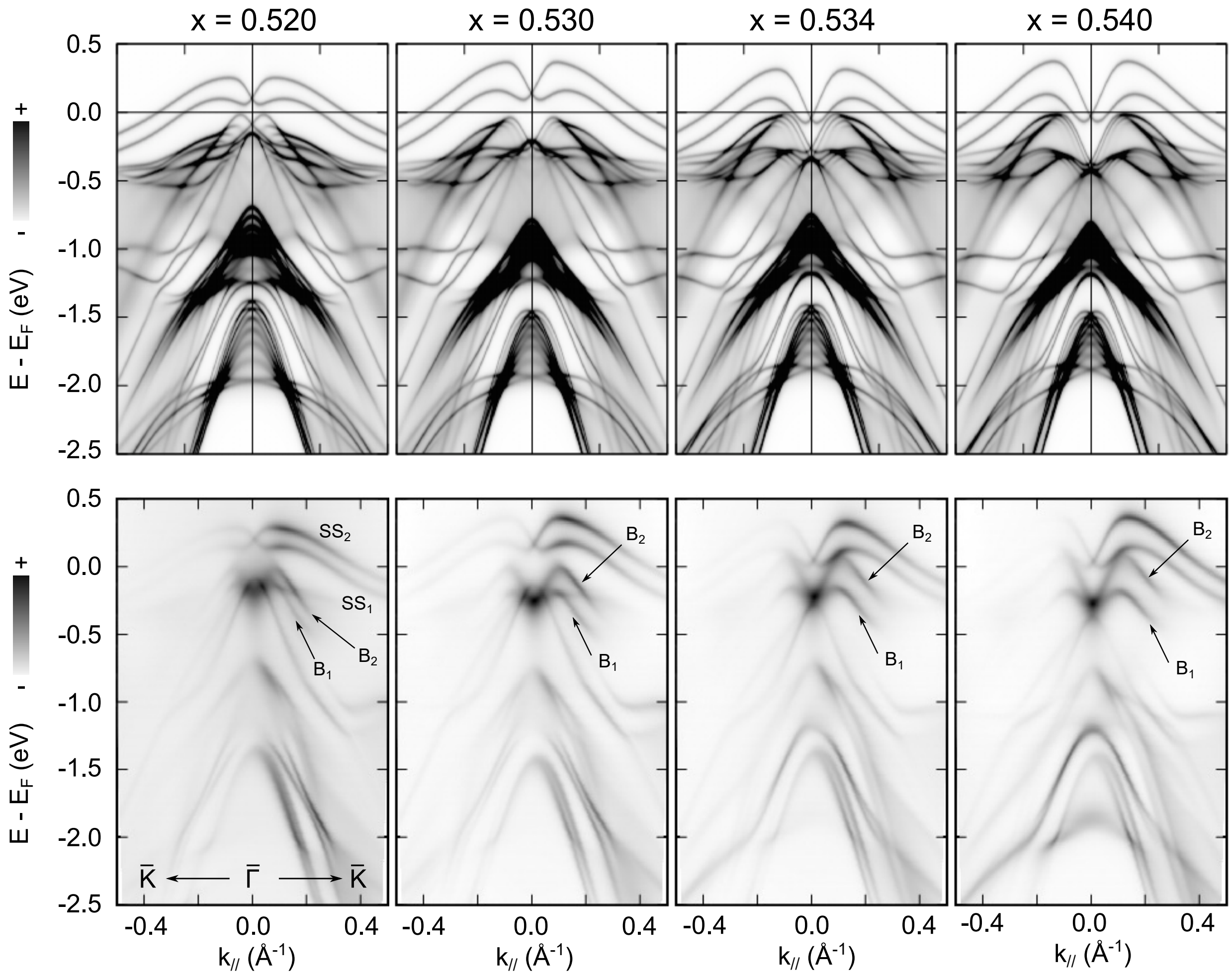

Supplementary Fig. 5: BSF (top) and ARPES (bottom) calculations of α-GeTe(111) for different x positions of the Te atom in the primitive unit cell, as defined in the inset of Fig. 3d. The ground state corresponds to x = 0.530.

We also performed BSF, as well as ARPES calculations using the one-step model of photoemission. The calculations are presented in Supplementary Fig. 5 and show the same evolution (as presented in Supplementary Fig. 4) of the Rashba splitting of the bulk states as a function of x.

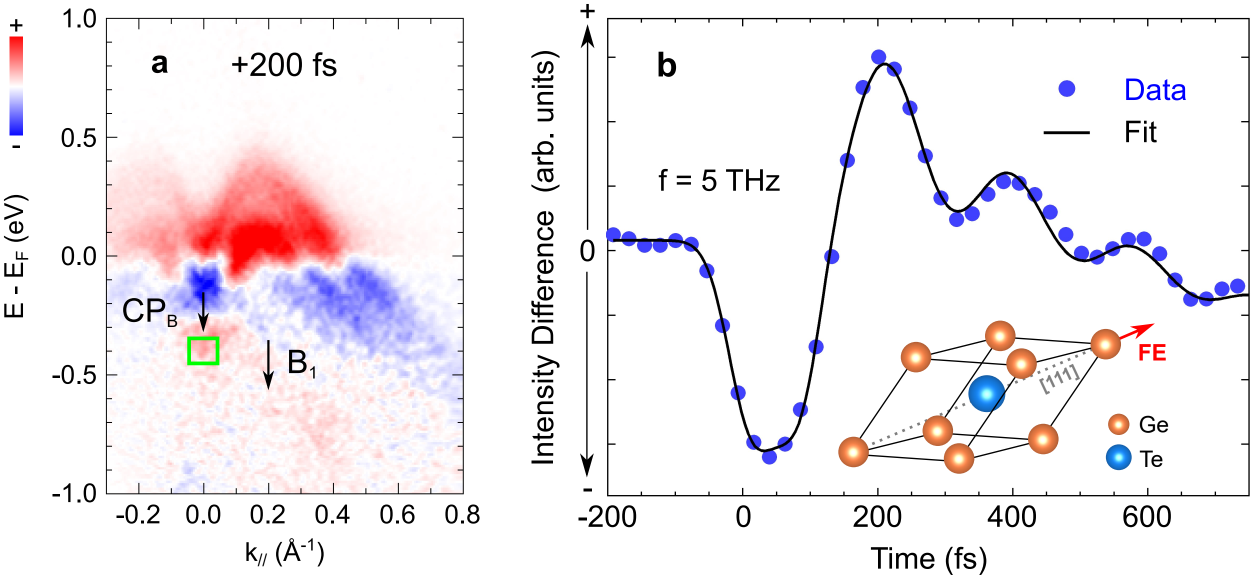

Supplementary Fig. 6: (a) +200 fs difference tr-ARPES intensity map. (b) Transient photoemission intensity within the green integration square indicated in panel (a) showing a coherent oscillation at a frequency of 5 THz.

Coherent A_{1g} phonons mode

Supplementary Fig. 6a shows the difference tr-ARPES map at +200 fs, corresponding to the maximum amplitude of delayed displacive excitation of a coherent phonon (DECP) mechanism as explained in the main text. In Supplementary Fig. 6b we plot the transient photoemission intensity within the green box in panel (a). It shows a coherent modulation with a 5 THz frequency as extracted from fitting procedure, in good agreement with our extraction of the transient evolution of the CP_B shift discussed in Fig. 3 of the main text. This coherent modulation is associated to the A_{1g} phonon mode, as evidenced by bulk phonon dispersions calculations at $\bar{\Gamma}$ in the FE phase.[2] At this high symmetry point a 4.4 THz frequency value is reported. The deviation with our measurements can be explained both by the difference of the subsurface structure with the bulk one and by the transient modification of the shape of the ion energy potential landscape.

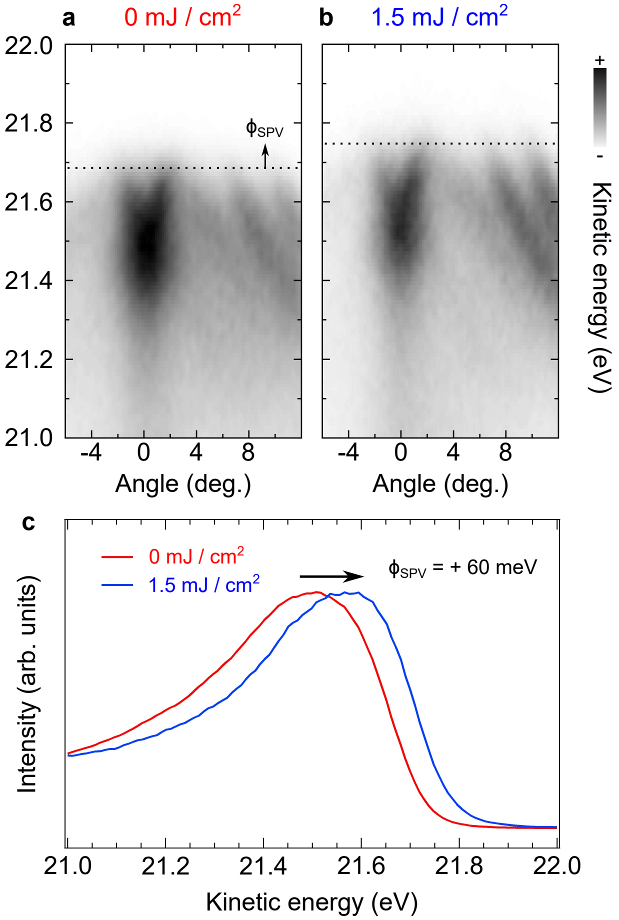

Supplementary Fig. 7: tr-ARPES measurements of α-GeTe(111) at negative pump-probe delay for an absorbed fluence of (a) 0 mJ/cm^2 and (b) of 1.5 mJ/cm^2. (c) Corresponding angle integrated EDCs. A positive SPV of +60 meV is observed due to the compensation of the downward BB as depicted in Supplementary Fig. 8. The kinetic energy has been corrected by the work function of the photoemission analyser.

Evidence of a positive surface photovoltage (SPV)

Supplementary Fig. 7 displays the tr-ARPES measurements of α-GeTe(111) at -1 ps pump-probe delay without (red) and with (blue) the pump. From this, it is possible to evaluate the magnitude and the sign of the SPV.[3,4] We observe that the blue spectrum is shifted by +60 meV to high kinetic energy with respect to the red one. A downward band bending (BB) is expected for p doped α-GeTe(111) with surface donor states, as illustrated in the top panel of Supplementary Fig. 8b. This is confirmed by the positive value of the SPV which is compensating the downward BB.

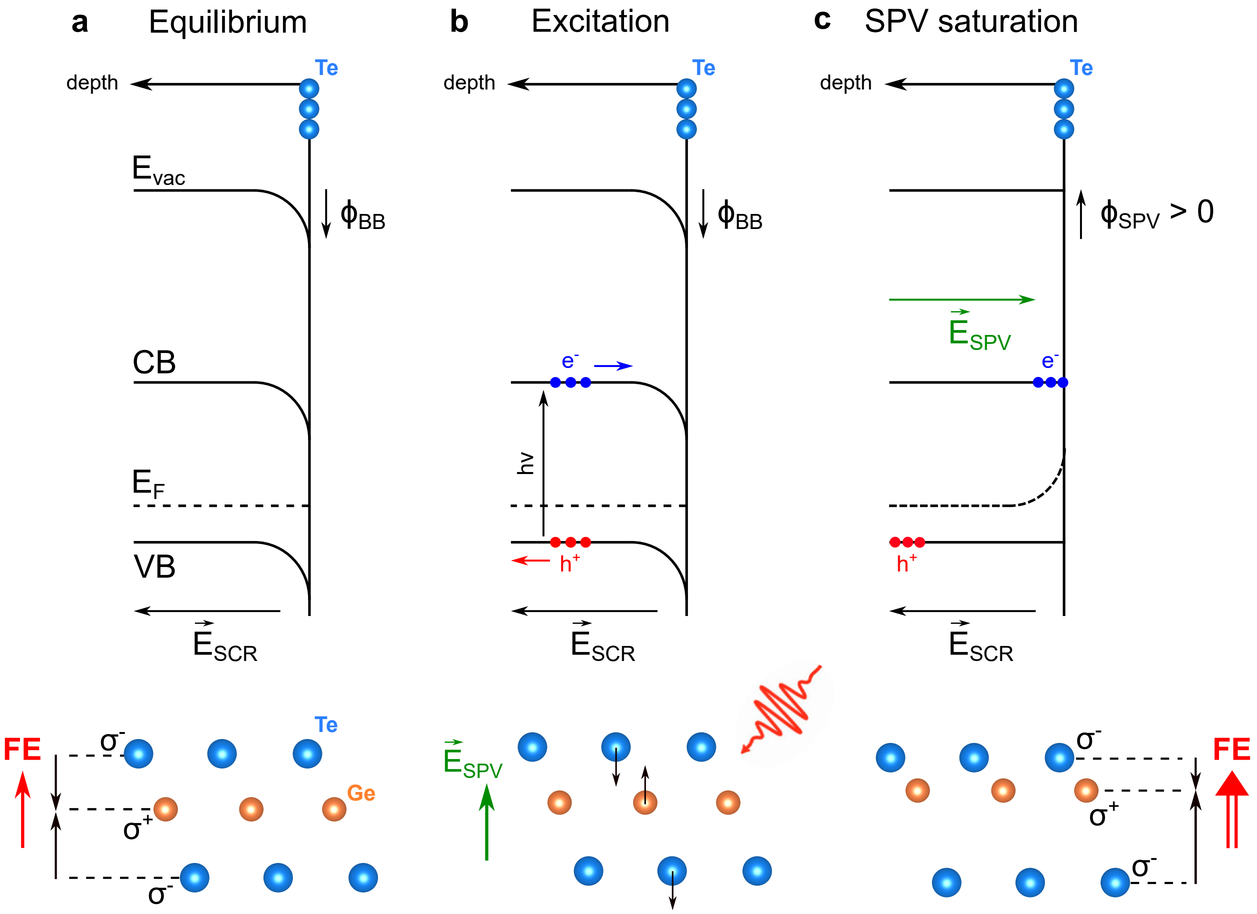

Supplementary Fig. 8: Schematic diagram of electron energy levels near the surface (top) and corresponding subsurface structure (bottom) of α-GeTe(111): (a) At the equilibrium, (b) under the illumination and (c) after the charge carriers redistribution and SPV saturation.

Downward BB and positive SPV in α-GeTe(111)

Supplementary Fig. 8 displays the energy band diagrams of a p-doped α-GeTe(111) surface. In each case, we consider the (a) nonequilibrium, (b) the equilibrium and the (c) photoexcited configurations. For a p-doped surface with surface donors states, a downward BB and consequently a positive SPV are expected. These expectations are confirmed by our measurements in Supplementary Fig. 7. In the particular case of a FE material, the generated compensative electric field (E_{SPV}) affects the initial surface dipole. In the present case, we expect that photoinduced E_{SPV} push Te atoms into the bulk and Ge atoms into the surface, leading to a photoinduced increase of the FE distortion: see bottom panels. In other

words, E_{SPV} reinforces the initial net dipole at the surface originating from the Te surface termination and short bonds between the first Te and the subsurface Ge planes.

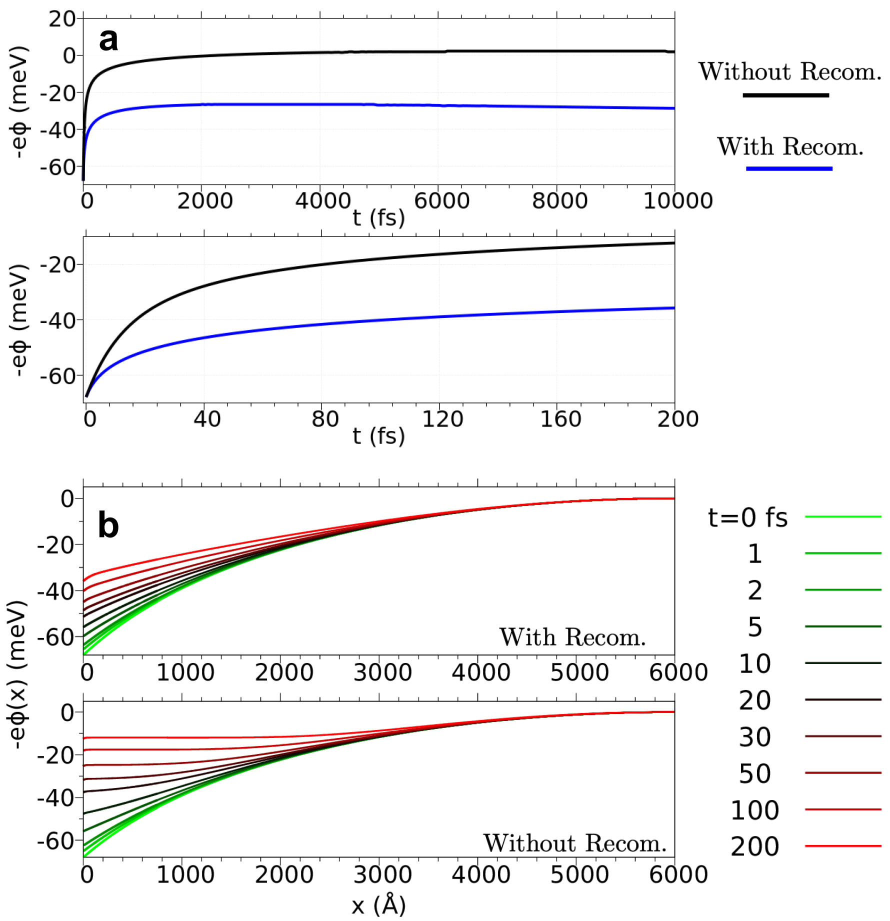

Supplementary Fig. 9: Non-equilibrium dynamics obtained by solving the drift-diffusion equations. (a) The SPV as a function of time after the pump for systems with recombination (blue) and without recombination (black). (b) The band energy as a function of position x at different times. The upper (lower) panel is for system with (without) recombination. The parameters used in the simulation are shown in Supplementary Table 1.

Simulation of the carrier dynamics in photoexcited GeTe

We apply the numerical procedure presented in Ref[4] to solve the drift-diffusion equations (DDEs) in combination with the Poisson equation, using the realistic parameters listed in Supplementary Table 1.

Let us first clarify the static situation before photo-doping. Since the surface states have a higher energy than the bulk states, they can dope electrons into bulk, which leaves holes confined in the surface states. We assume that the holes remain trapped near the surface with an exponentially decaying density profile $N_d(x) = n_u \lambda_d e^{-\lambda_d \frac{x}{l_0}}$, where $l_0 = 1$ is the unit

Supplementary Table 1: Parameters used in the simulation. The diffusion coefficients are obtained by the Einstein relation $D_{n,p} = \frac{\mu_{n,p} k_B T}{\mathrm{e}}$. The values of μ_n, μ_p, ε_r are from Ref.[5,6] The coefficient α in B_r is a unit-less number. We choose $\alpha = 0$ and $\alpha = 0.3$ in the simulation.

Name	Symbol	Value
Temperature	T	300K
Relative Permittivity	ε_r	36.0
Electron Mobility	μ_n	$100.0 \times 10^{-4}\mathrm{m}^2/(\mathrm{Vs})$
Electron Diffusion Coefficient	D_n	$2.585 \times 10^{-4}\mathrm{m}^2/\mathrm{s}$
Hole Mobility	μ_p	$100 \times 10^{-4}\mathrm{m}^2/(\mathrm{Vs})$
Hole Diffusion Coefficient	D_p	$2.585 \times 10^{-4}\mathrm{m}^2/\mathrm{s}$
Total surface density of holes	σ_0	$8.0 \times 10^{14}\mathrm{m}^{-2}$
Photo-excited Carrier Volume	σ_p	$\sim 1399\sigma_0$
Length Unit	l_0	$10^{-10}\mathrm{m}$ (1Å)
Sample Length	L	$6000 \cdot l_0$
Number of x-grid points	N	30001
Electric Field Unit	$E_u = \frac{\sigma_0 e}{2\varepsilon_r \varepsilon_0}$	$2.011 \times 10^5\mathrm{V/m}$
Density Unit	$n_u = \frac{\sigma_0}{l_0}$	$8.0 \times 10^{24}\mathrm{m}^{-3}$
Radiative Recombination Rate	$B_r = \frac{\alpha}{10\mathrm{fs} \cdot n_u}$	$1.25\alpha \times 10^{-11}\mathrm{m}^3/\mathrm{s}$

of length, σ_0 the total surface density of holes, $n_u = \sigma_0/l_0$ the unit of density. We choose $\lambda_d = 1/5$, which means the density of holes decays within 5Å to the bulk. We determine $\sigma_0 = 8.0 \times 10^{14} m^{-2}$ using the information on the band bending in equilibrium. The positive charge from this hole distribution will lead to an accumulation of electrons near the surface. However, because of the large relative permittivity ε_r and large diffusion coefficients, the free electrons distribution $n(x)$ decays much more slowly into the bulk. Charge neutrality requires $\int N_d(x) = \int n(x) \equiv \sigma_0$. In static state, there are more holes near surface and more free electrons away from the surface. This electron-hole separation creates a space charge region, with a net electric field E pointing to the bulk. We find the maximum value of $E \approx 2E_u$, with $E_u = 2.011 \times 10^5\mathrm{V/m}$.

We assume that the photo-excited electron-hole pairs are generated instantaneously at $t = 0_+$ with the density profile $\delta n(x, t = 0_+) = \delta p(x, t = 0_+) = \gamma_0 e^{-x/\lambda_{ph}}$, where $\lambda_{ph} = 80$ nm is penetration depth and γ_0 the photo-excited carrier (surface) density with $\gamma_0 \approx 1399\sigma_0 \gg \sigma_0$. To take inter-band recombination into account, we introduce an ad-hoc term $B_r \delta_n \delta_p$. The

DDEs are solved for systems at $T = 300$K and the results are shown in Supplementary Fig. 9. The SPV ϕ_{SPV} as a function of t is plotted in Supplementary Fig. 9a, with the upper panel showing the time range $0 < t < 10000$ fs and the lower panel a zoom of the time range $0 < t < 200$ fs. Upon the photo-doping, we find a fast change in ϕ_{SPV} in first 10 fs, but it takes much longer time $\sim$ 100 fs to reach half the maximum change in ϕ_{SPV}. This is much slower than the time scale $\sim$ 1 fs obtained for black phosphorus (BP),[4] since the drift mobility in GeTe is around $v = \mu E \sim 0.04$ Å/fs, which is nearly 250 times smaller than that ($\sim$ 10 Å/fs) in BP. The corresponding energy band -e$\phi(x)$ is shown in Supplementary Fig. 9b. These data show that the internal electric field is weakened in the photo-doped systems (flattening of the energy bands), which is much more evident in system without recombination.

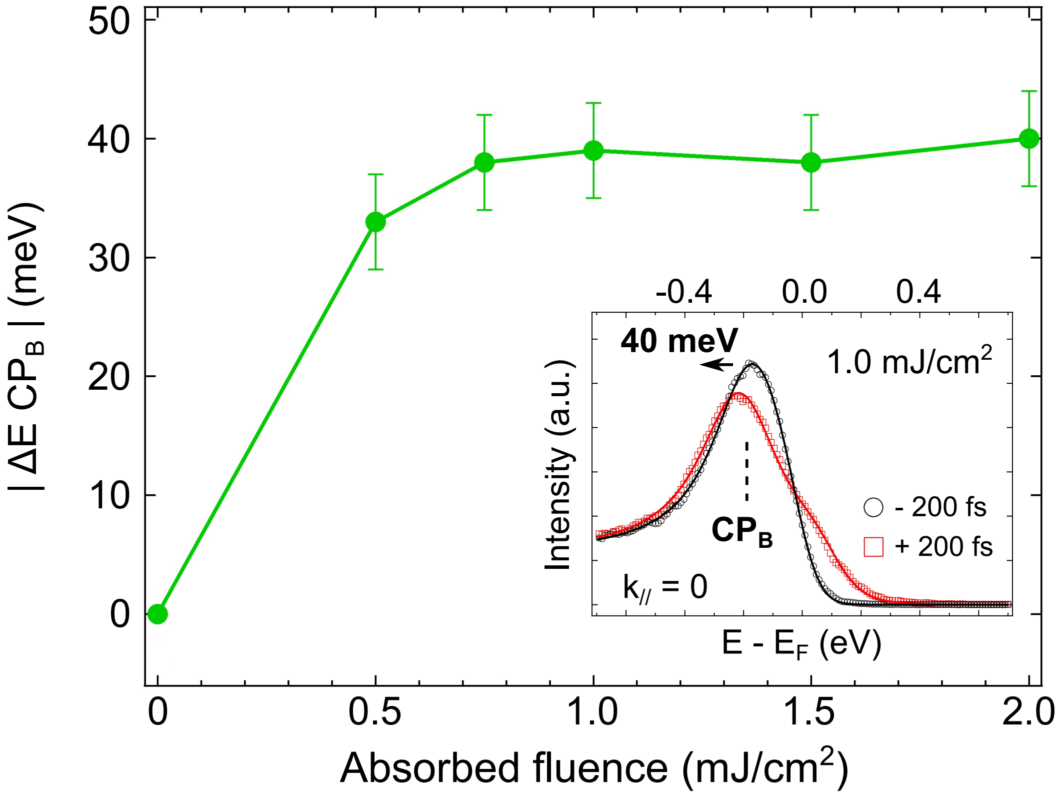

Supplementary Fig. 10: Absorbed fluence dependence of the photoinduced shift of the CP_B at +200 fs as extracted from EDCs at the $\bar{\Gamma}$ point : see inset.

Fluence dependence

Supplementary Fig. 10 displays the fluence dependence of the CP_B at +200 fs. It shows a rapid saturation at a 0.5 mJ/cm^2 treshold. This is unexpected in a standard DECP picture but can be understood in our delayed DECP mechanism invoking SPV. Indeed, it is well documented that SPV quickly saturates as a function of the fluence.[7]

Supplementary References

1. Sobota, J. A.; Yang, S.; Analytis, J. G.; Chen, Y. L.; Fisher, I. R.; Kirchmann, P. S.; Shen, Z.-X. Ultrafast Optical Excitation of a Persistent Surface-State Population in the Topological Insulator Bi_2Se_3. *Phys. Rev. Lett.* **2012**, *108*, 117403.

2. Dangić, D.; Hellman, O.; Fahy, S.; Savić, I. The origin of the lattice thermal conductivity enhancement at the ferroelectric phase transition in GeTe. *Npj Comput. Mater.* **2021**, *7*, 1–8.

3. Yang, S.-L.; Sobota, J. A.; Kirchmann, P. S.; Shen, Z.-X. Electron propagation from a photo-excited surface: implications for time-resolved photoemission. *Appl. Phys. A* **2014**, *116*, 85–90.

4. Kremer, G.; Rumo, M.; Yue, C.; Pulkkinen, A.; Nicholson, C. W.; Jaouen, T.; von Rohr, F. O.; Werner, P.; Monney, C. Ultrafast dynamics of the surface photovoltage in potassium-doped black phosphorus. *Phys. Rev. B* **2021**, *104*, 035125.

5. Tsu, R.; Howard, W. E.; Esaki, L. Optical and Electrical Properties and Band Structure of GeTe and SnTe. *Phys. Rev.* **1968**, *172*, 779–788.

6. Valassiades, O.; Economou, N. Hole mobility in GeTe single crystals. *Phys. Lett.A* **1977**, *63*, 133–135.

7. Chen, Z.; Dong, J.; Giorgetti, C.; Papalazarou, E.; Marsi, M.; Zhang, Z.; Tian, B.; Ma, Q.; Cheng, Y.; Rueff, J.-P., *et al.* Spectroscopy of buried states in black phosphorus with surface doping. *2D Mater.* **2020**, *7*, 035027.
