# Supplementary material for: Field-induced ultrafast modulation of Rashba coupling at room temperature in ferroelectric α-GeTe(111)
Source: Nat Commun. 2022 Oct 27;13:6396. doi: 10.1038/s41467-022-33978-3 (PMC9613697; doi:10.1038/s41467-022-33978-3)
Supplement: Supplementary file 3 — Description of Additional Supplementary Files [file 41467_2022_33978_MOESM3_ESM.pdf]

Description of Additional Supplementary Files

Supplementary Software 1 In this supplementary .zip file, the reader can find a custom code based on the numerical solving of the drift-diffusions equations to simulate the non-equilibrium dynamics of photo-excited carriers in open-boundary semiconductors or semimetals.
