# Supplementary material for: Field-induced ultrafast modulation of Rashba coupling at room temperature in ferroelectric α-GeTe(111)
Source: Nat Commun. 2022 Oct 27;13:6396. doi: 10.1038/s41467-022-33978-3 (PMC9613697; doi:10.1038/s41467-022-33978-3)
Supplement: Supplementary file 4 — Supplementary Software 1 [file 41467_2022_33978_MOESM4_ESM.zip › pump_drift_diffusion_code_changming_yue/pump_drift_diffusion_code_changming_yue/Readme_drift_diffusion_simulation.pdf]

pump_drift_diffusion: A drift-diffusion simulation of the non-equilibrium dynamics of photo-excited carriers

Author : Changming Yue
University of Fribourg, Switzerland
Date : 22/08/2022

1. Overview

This package is developed by Dr. Changming Yue from University of Fribourg, Switzerland. It simulates the non-equilibrium dynamics of photo-excited carriers in open-boundary semiconductors or semimetals, for example black phosphorus [1] and GeTe [2], by numerically solving the drift-diffusion equations with an ad-hoc recombination process. The implementation follows the appendix of Ref. [1]. The outputs include (1) the band energy $-e\phi(x,t)$ as a function of position x and time t, with the value at $-e\phi(x=0,t)$ being the surface photovoltage. (2) The net electrons density $\delta n(x,t)$, net holes density $\delta p(x,t)$, and internal electric field $E(x,t)$ as a function of position x and time t.

References:

[1] Phys. Rev. B 104, 035125 (2021)

[2] arXiv.2204.11630

2. System Requirements

• Hardware Requirements

This package requires only a standard computer with enough RAM (recommend RAM>4GB).

• OS Requirements

Linux (tested only in CentOS Linux)

3. Software Requirements

Python 2.7, Spicy ⩾1.2.2, numpy ⩾ 1.16, gnuplot 5.4

4. Installation Guide

No additional steps are needed as long as the system and software requirements are satisfied.

5. Examples and usage:

There are two examples inside the folder of “example”: “no_recombination” and “recombination”. They correspond to $\alpha=0$ and $\alpha=0.3$, respectively, in the formula of radiative recombination rate in the Table 1 of the supplementary material of Ref [2].

Please enter each subfolder and run the job script “run_spr.sh”. It usually takes several days to finish the calculation. However, you can kill the job if the simulation time is enough long by checking the output file “dde.out” by “tail dde.out”.

You can do the post-processing during or after the simulation by running the script “post_phi_x_phix0.sh” in each subfolder.

Once the post-processing is done, you can

(1) Plot the surface photo-voltage in the home folder of “example” by running “gnuplot plot_surface_photovoltage.plt” with the output figures “surface_photovoltage_long_time.png” and “surface_photovoltage_short_time.png”, which should reproduce the panel (a) in Fig. S9.

(2) Plot the band energy as a function of x and t by running “gnuplot plot_band_energy_xt.plt” with the output figure “bandenergy_xt.eps”, which should reproduce the panel (b) in Fig. S9.
