# Supplementary figures and images for: Field-induced ultrafast modulation of Rashba coupling at room temperature in ferroelectric α-GeTe(111)
Source: Nat Commun. 2022 Oct 27;13:6396. doi: 10.1038/s41467-022-33978-3 (PMC9613697; doi:10.1038/s41467-022-33978-3)

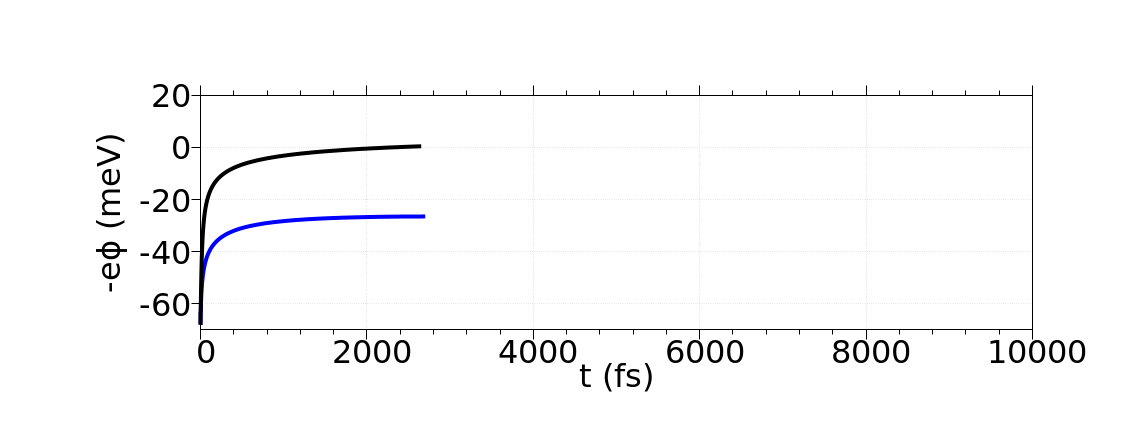

Supplement: Supplementary file 4 — Supplementary Software 1 [file 41467_2022_33978_MOESM4_ESM.zip › pump_drift_diffusion_code_changming_yue/pump_drift_diffusion_code_changming_yue/example/surface_photovoltage_long_time.png]

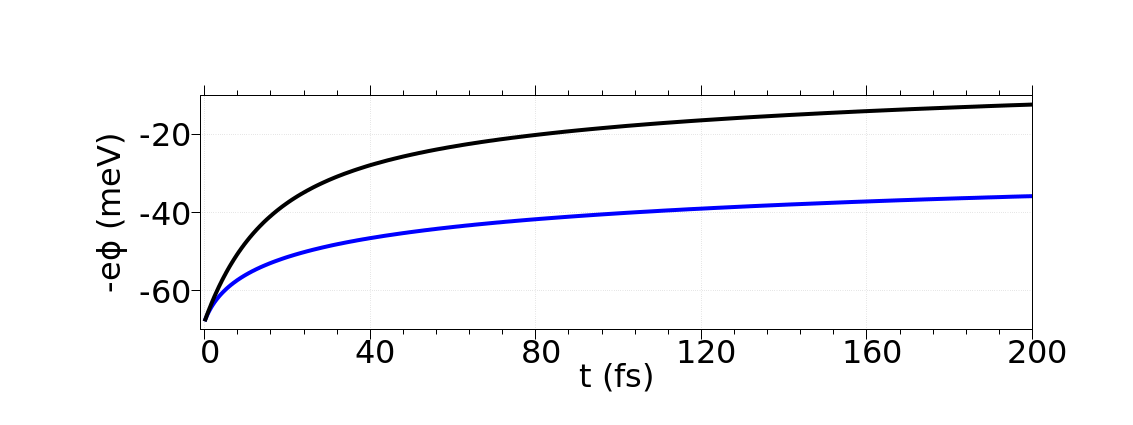

Supplement: Supplementary file 4 — Supplementary Software 1 [file 41467_2022_33978_MOESM4_ESM.zip › pump_drift_diffusion_code_changming_yue/pump_drift_diffusion_code_changming_yue/example/surface_photovoltage_short_time.png]
