# Supplementary material for: Field-induced ultrafast modulation of Rashba coupling at room temperature in ferroelectric α-GeTe(111)
Source: Nat Commun. 2022 Oct 27;13:6396. doi: 10.1038/s41467-022-33978-3 (PMC9613697; doi:10.1038/s41467-022-33978-3)
Supplement: Supplementary file 5 — Lasing Reporting Summary [file 41467_2022_33978_MOESM5_ESM.pdf]

nature research

Corresponding author(s): Geoffroy Kremer & Jan Minar

Lasing Reporting Summary

Nature Research wishes to improve the reproducibility of the work that we publish. This form is intended for publication with all accepted papers reporting claims of lasing and provides structure for consistency and transparency in reporting. Some list items might not apply to an individual manuscript, but all fields must be completed for clarity.

For further information on Nature Research policies, including our data availability policy, see Authors & Referees.

~ Experimental design

Please check: are the following details reported in the manuscript?

1. Threshold

Plots of device output power versus pump power over a wide range of values indicating a clear threshold

- [ ] Yes — N/A
- [ ] No — no laser development ; see Ref. 16 and other therein

2. Linewidth narrowing

Plots of spectral power density for the emission at pump powers below, around, and above the lasing threshold, indicating a clear linewidth narrowing at threshold

- [ ] Yes — N/A
- [ ] No — no laser development ; see Ref. 16 and other therein

Resolution of the spectrometer used to make spectral measurements

- [ ] Yes — N/A
- [ ] No — no laser development ; see Ref. 16 and other therein

3. Coherent emission

Measurements of the coherence and/or polarization of the emission

- [ ] Yes — N/A
- [ ] No — no laser development ; see Ref. 16 and other therein

4. Beam spatial profile

Image and/or measurement of the spatial shape and profile of the emission, showing a well-defined beam above threshold

- [ ] Yes — N/A
- [ ] No — no laser development ; see Ref. 16 and other therein

5. Operating conditions

Description of the laser and pumping conditions
Continuous-wave, pulsed, temperature of operation

- [x] Yes — in the methods section
- [ ] No

Threshold values provided as density values (e.g. W cm^{-2} or J cm^{-2}) taking into account the area of the device

- [x] Yes — in the figure caption for the corresponding measurements
- [ ] No

6. Alternative explanations

Reasoning as to why alternative explanations have been ruled out as responsible for the emission characteristics
e.g. amplified spontaneous, directional scattering; modification of fluorescence spectrum by the cavity

- [ ] Yes — N/A
- [ ] No — no laser development ; see Ref. 16 and other therein

7. Theoretical analysis

Theoretical analysis that ensures that the experimental values measured are realistic and reasonable
e.g. laser threshold, linewidth, cavity gain-loss, efficiency

- [ ] Yes — N/A
- [ ] No — no laser development ; see Ref. 16 and other therein

8. Statistics

Number of devices fabricated and tested

- [ ] Yes — N/A
- [ ] No — no new laser device developped here

March 2018

Statistical analysis of the device performance and lifetime (time to failure)	☐ Yes	N/A
	☐ No	no laser development ; see Ref. 16 and other therein
